# The effect of type 2 diabetes on periapical osteoclast-related factors during axial tooth movement

**DOI:** 10.3389/fendo.2025.1637624

**Published:** 2025-08-28

**Authors:** Jing Shi, Tong Lin, Ziqing Shi, Jiaoyang Zhao, Xingqi An, Yao Xu, Li Zhu, Wenjin Li

**Affiliations:** ^1^ The Second Hospital of Tianjin Medical University, Tianjin, China; ^2^ Shanxi Medical University School and Hospital of Stomatology, Taiyuan, China; ^3^ The Second Clinical Medical Department of Shanxi Medical University, Taiyuan, China

**Keywords:** type 2 diabetes, osteoclast, axial tooth movement, RANK, RANKL, OPN

## Abstract

**Objectives:**

To explore the impact of type 2 diabetes on tooth axial elongation and its relationship with osteoclast-related factors.

**Materials and methods:**

We established an unopposed molars model of type 2 diabetic mice, and recorded changes in mandibular bone mineral density (BMD) using micro-CT. Changes in the cells and fibers were observed by Hematoxylin and Eosin (HE) staining, Masson staining and Tartrate Resistant Acid Phosphatase (TRAP) activity assay of the right mandibles. The expression levels of osteopontin (OPN), receptor activator for nuclear factor-κ (RANK) and Receptor Activator of Nuclear Factor-κ B Ligand (RANKL) were observed using immunohistochemistry and RT-qPCR.

**Results:**

Micro-computed tomography (CT) analysis showed that tooth elongation and bone mineral density (BMD) in both groups increased over time but were consistently lower in diabetic mice compared to controls. Histological staining showed that diabetic mice had more osteoclasts and bone resorption, with sparser collagen. Immunohistochemistry and RT-qPCR showed that the expression levels of osteopontin (OPN), receptor activator for nuclear factor-κ (RANK), and receptor activator of nuclear factor-κ B ligand (RANKL) in both groups increased over time but were higher in diabetic mice compared to controls.

**Conclusions:**

Type 2 diabetes may slow down the axial elongation of teeth. The aggravate bone resorption based on the abnormal expression of RANK, RANKL, and OPN was a probable reason for the inhibition of alveolar bone remodeling.

## 1 Introduction

Tooth movement is a dynamic process that occurs throughout life—during tooth eruption, orthodontic treatment, periodontitis, and other pathologic and physiological processes (1–3). Currently, studies mainly focus on horizontal tooth movement (4, 5). This primarily pertains to the adjustment made during orthodontic procedures, that is, with human intervention, involving the remodeling of the alveolar bone and the response of periodontal tissues triggered by external forces (6). These forces are different from the biomechanics in the process of natural tooth growth and guide the teeth towards a specific direction; thus, it is a passive movement process influenced by many external factors (7). The research indicates that such adjustments during orthodontic processes may amplify bacteria-induced periodontal inflammation (8). Moreover, orthodontic treatment causes root resorption, and above the initial stress threshold, the degree of root resorption increases over time (9). Therefore, this process cannot truly reflect the natural process of dental growth.

Another manner of tooth movement involves axial change. This includes tooth eruption, the elongation of tooth in the opposite jaw after tooth loss, and pathological axial tooth migration. Such change is consistent with the direction of tooth eruption and can occur naturally without external forces. This process is a normal aspect of dental development, which is crucial for both the functionality and aesthetics of teeth. In prosthodontics, this phenomenon is important for establishing occlusal relationships and designing dentures (10). Research has shown that axial tooth movement is the result of alveolar bone remodeling (11). During tooth eruption, the bone over the tooth crown demonstrates bone absorption traits, leading to the formation of vesicles marked by osteoclasts, while the bone beneath the tooth develops into bone trabeculae that are covered by osteoblasts (12). In addition, failure in the physiologic process is closely linked to reduced bone resorption (13). Osteoclasts, the primary cells responsible for bone resorption, are crucial in this process.

The function of osteoclasts is affected by numerous factors (14). Diabetes is a chronic and progressive disorder of the endocrine system, arising from anomalies in insulin production or functional impairments (15). Hyperglycemia can lead to metabolic abnormalities and inflammatory environment, potentially causing a range of severe complications, including adverse effects on bone metabolism factors and various disorders of the skeletal system (16). It has the potential to reduce the production of reactive oxygen species through anti-oxidation mechanisms, and can affect the activity of caspase-3, as well as disrupt nuclear factor kappa B (NF-κB) receptor activator ligand signaling, thereby enhancing osteoclast activity and proliferation (17). Therefore, inhibiting the formation of osteoclasts and the process of bone resorption is a target for therapeutic intervention. Receptor activator of nuclear factor-κ B (RANK) and receptor activator of nuclear factor-κ B ligand (RANKL) are members of the tumor necrosis factor (TNF) superfamily (18, 19). RANKL is highly expressed in osteocytes (19), and RANK is primarily found on the surface of osteoclast precursor cells, osteoclasts, and lymphocytes (18). As a membrane-associated cytokine, RANKL induces osteoclast differentiation and reduces osteoclast apoptosis by binding to RANK (20, 21). Osteopontin (OPN), as an extracellular matrix protein, can promote the expression of RANKL and accelerate bone resorption (22). Currently, research on OPN primarily focuses on the growth and development of teeth and the reconstruction of dental and periodontal tissues (23, 24); studies on RANK and RANKL focus on their roles in bone metabolism, immune system regulation, and tumor biology (18). However, few research has focused on their role in axial tooth movement.

In this study, we observed the axial elongation of teeth in the unopposed molars model of type 2 diabetic mice and analyzed the related pathological and molecular mechanisms based on the abnormal expression of osteoclast-related factors.

## 2 Materials and methods

### 2.1 Ethics approval and inclusion to participate

The present work was approved by the Ethics Committee of Shanxi Medical University (ethical number: DW2022038), and all experiments were performed in accordance with relevant guidelines and regulations and ARRIVE guidelines.

### 2.2 Induction of the type 2 diabetic mouse model

A total of 150 C57BL/6J mice (6 weeks old, male, 20–25 g) were purchased from the Animal Laboratory Center of Shanxi Medical University. All the mice were fed adaptively for 2 weeks with a 12-h light/dark cycle at a temperature of approximately 23 °C. The living environment of mice was disinfected and the padding was changed for an average of 3 days. A total of 150 mice were randomly divided into the diabetes group (*n* = 80) and the control group (*n* = 70). The mice in the diabetes group were intraperitoneally injected with 40 mg/kg streptozotocin (STZ) dissolved in 0.1 mmol/L citrate buffer at pH 4.2 for five consecutive days. The mice in the control group were injected with the same dose of citrate buffer. Blood glucose (tail blood) was measured at 3 days after injection, and then once a week for 4 weeks. The mice were observed to have experienced polydipsia, polyphagia, and polyuria; the padding had obvious odor and moisture; and the fasting blood glucose was >11.1 mmol/L for 4 weeks, indicating that the diabetes mellitus model was established successfully.

### 2.3 Construction of the unopposed molars model

After the successful establishment of the type 2 diabetes mellitus model, the padding was changed and the living environment was disinfected every day, the mice in both groups were anesthetized with 1% pentobarbital sodium (50 mg/kg), all right maxillary molars were extracted, and hemostasis was achieved by compression.

### 2.4 Mandibular molar elongation and mandibular bone density analyses by micro-CT

After tooth extraction, mice were randomly divided into five subgroups: 0, 3, 6, 9, and 12 days. The mice were sacrificed using an excess of isoflurane anesthesia at corresponding days. Isoflurane (5% concentration) was poured onto a cotton ball inside an anesthesia chamber. The mice were placed in the chamber, and the lid was secured to prevent the isoflurane from escaping. The mice’s reactions were observed, and they typically lost consciousness within 3 min. After the mice lost consciousness, they were continued to be exposed to isoflurane for an extended period to ensure death. The right mandibular tissue of the mice was harvested. Ten mandibles from each group were selected and three-dimensional scanning was performed with the viva CT80 micro-computed tomography (CT) scanner at 70 kV and 114 μA at a resolution of 35 μm at 0, 3, 6, 9, and 12 days after tooth extraction. The line connecting the outermost tangent points above the bilateral mandibular foramen of mice was taken as the reference line, and a parallel line was made from the highest point of the cusp of the right mandibular first molar. The distance between the two parallel lines was measured at 3, 6, 9, and 12 days after tooth extraction, and the distance between the two parallel lines was subtracted at 0 days after tooth extraction to determine tooth elongation. The measurement was repeated three times and the average value to obtain the elongation of the mandibular molar was determined. The bone mineral density (BMD) of periapical tissue of mandibular molars was measured using CT analyzer V1.14.4, and the average value was determined.

### 2.5 Tissue preparation and histological analysis

The right mandible in each group of mice was fixed in 4% polyformaldehyde for 48 h, flushed with running water for 1 h, and decalcified with 10% EDTA for 45–50 days, and then the specimens were dehydrated, transparent, waxed, and embedded. The samples were cut into 4-mm sagittal sections along the long axis of the molar teeth. RANK antibody (A01064-1), RANKL antibody (K008348P), and OPN antibody (BM4208) were used to observe the changes of RANK, RANKL, and OPN in the periapical tissue of teeth by immunohistochemical staining. Statistical analysis of the positive area was performed using ImageJ software. The sections were stained with hematoxylin and eosin (HE), Masson, and tartrate-resistant acid phosphatase (TRAP). HE staining is used to distinguish dental tissue, general bone tissue structure, and cells. Masson staining can reflect the morphology of collagen fibers in periodontal ligament. TRAP enzyme histochemistry is used to evaluate osteoclasts. The samples were fixed with pre-cooled TRAP fixative for 60 s. After rinsing with distilled water, TRAP incubation solution was applied to cover the sections and then it was incubated in a humidified chamber at 37 °C for 50 min. This was followed by rinsing with distilled water, counterstaining with methyl green for 2 min, rinsing again with distilled water, removing excess moisture, and applying an aqueous mounting medium before microscopic examination.

### 2.6 Gene expression analysis by real-time reverse transcription polymerase chain reaction

Mandibular bone tissue was ground into powder using liquid nitrogen and transferred into a centrifuge tube. Trizol was added for RNA extraction. The tube was vortexed and centrifuged, and the supernatant was collected. Chloroform was added to the supernatant, shaken until the solution was clear and separated, then left to stand at room temperature for 10 min. After centrifugation, the supernatant was collected, and an equal volume of isopropanol was added. The mixture was left at room temperature for another 10 min before centrifugation. The white precipitate at the bottom of the tube was the extracted RNA. The RNA concentration and the OD_260_/OD_280_ ratio were measured using a NanoDrop spectrophotometer. The concentration was calculated using the following formula: RNA concentration (μg/μL) = 0.04 × (OD_260_ − OD_280_) × dilution factor. An OD_260_/OD_280_ ratio of approximately 1.8–2.2 indicated good RNA purity. The extracted RNA was reverse transcribed into complementary cDNA using PrimeScript RT Master Mix. PCR amplification was performed using the TB Green™ Premix Ex Taq™ II kit with the following conditions: 95 °C for 30 s (one cycle), followed by 95 °C for 5 s and 60 °C for 30 s (40 cycles). 18s was used as the reference gene. The CT values obtained from the analysis software were processed using the 2^−ΔΔCt^ method, and statistical analysis was performed using *t*-tests. The expression levels of the RANK, RANKL, and OPN genes were measured in both the diabetic and control groups, and the expression of these genes on days 3, 6, 9, and 12 was compared to that on day 0. The primer sequences for RANK, RANKL, and OPN are shown in **Table 1**.

**Table 1:** Primer sequences used for real-time PCR.

Genes	Forward primer (5′–3′)	Reverse primer (5′–3′)
RANK	TTCGACTGGTTCACTGCTCC	CCTCAGAATCCACCGTGCTT
RANKL	TCCTGTACTTTCGAGCGCAG	CCTGCAGGAGTCAGGTAGTG
OPN	ATGATGGCCGAGGTGATAGT	ACCATTCAACTCCTCGCTTT
18 s	CGGCTACCACATCCAAGGGAA	GCTGGAATTACCGAGGC

### 2.7 Statistical analysis

SPSS 25.0 statistical software was used for statistical processing. All data are expressed as the mean ± standard deviation, which conformed to the normal distribution and has homogeneous variance. *t*-test was used for all data. *p* < 0.05 was considered statistically significant.

## 3 Results

### 3.1 The unopposed molars model of type 2 diabetic mice

The type 2 diabetic mouse model was successfully established in 72 mice, with a success rate of 90%. The failure was caused by two mice dying from excessive injection of STZ into the blood, three mice dying from hyperglycemia infection, and three mice suffering from substandard blood glucose. A total of 70 mice were selected as experimental samples. All the mice in both groups successfully made up the unopposed molars model.

### 3.2 Comparison of the elongation and bone mineral density of right mandibular molars between the two groups

**Figure 1:**
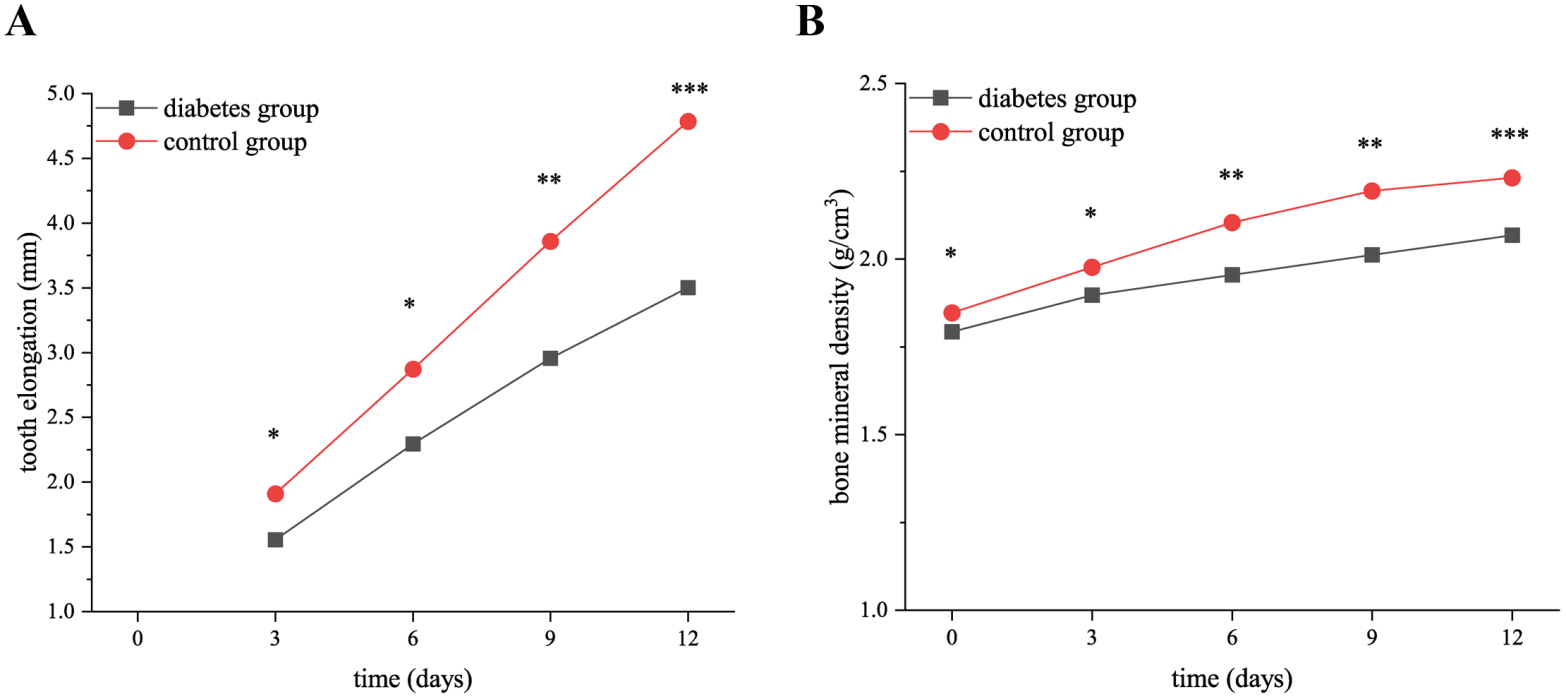
The elongation of right mandibular molars and the changes in bone mineral density in the diabetes and control groups. **(A)** shows the elongation of right mandibular molars in the diabetes and control groups at 0, 3, 6, 9, and 12 days after tooth extraction. The elongation of molars in both groups increased gradually over time, and the elongation of the diabetes group was less than that of the control group. **(B)** presents the BMD in the diabetes and control groups at 0, 3, 6, 9, and 12 days after tooth extraction. The BMD of both groups increased over time, and the increase in BMD of the diabetes group was significantly lower than that of the control group. .

The micro-CT showed that the elongation of right mandibular molars in both groups increased gradually with the extension of tooth extraction time, and the elongation of the diabetes group was significantly less than that of the control group (3 days: *p* = 0.01, 6 days: *p* = 0.01, 9 days: *p* = 0.002, 12 days: *p* < 0.001), as shown in **Figure 1A**. During the axial elongation of the right mandibular molar of mice, the density of the right mandible increased in both groups, and the change in the BMD of diabetes mice was significantly less than that of the control group (0 days: *p* = 0.03, 3 days: *p* = 0.02, 6 days: *p* = 0.003, 9 days: *p* = 0.002, 12 days: *p* < 0.001), as shown in **Figure 1B**.

### 3.3 Histological staining discrepancies in right mandibles of the diabetes group versus the control group

#### 3.3.1 Immunohistochemical staining of right mandibles

**Figure 2:**
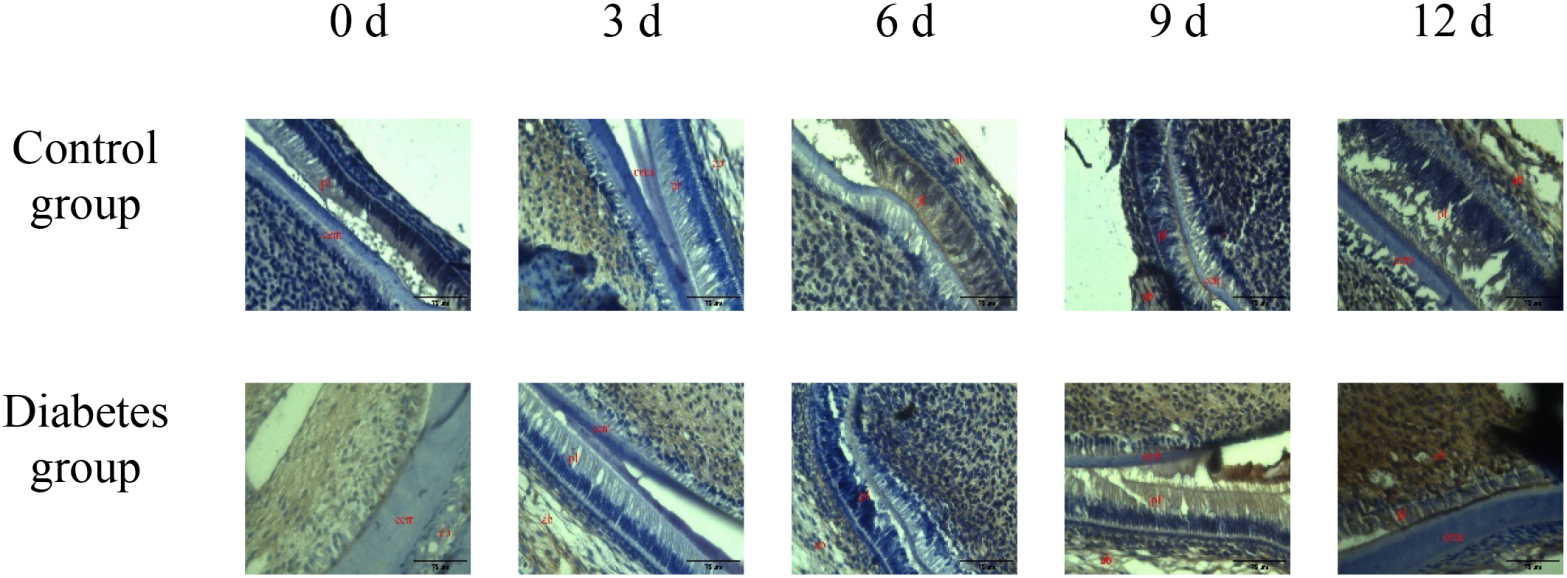
The expression of OPN in periapical tissues of the diabetes group and control group. Immunohistochemical staining showed that OPN had obvious brown staining in the alveolar bone absorption area. OPN protein expression was strongly positive in the diabetes group, and a weak positive staining was observed in the control group. cem, cementum; ab, alveolar bone; pl, periodontal ligament (×20).

**Figure 3:**
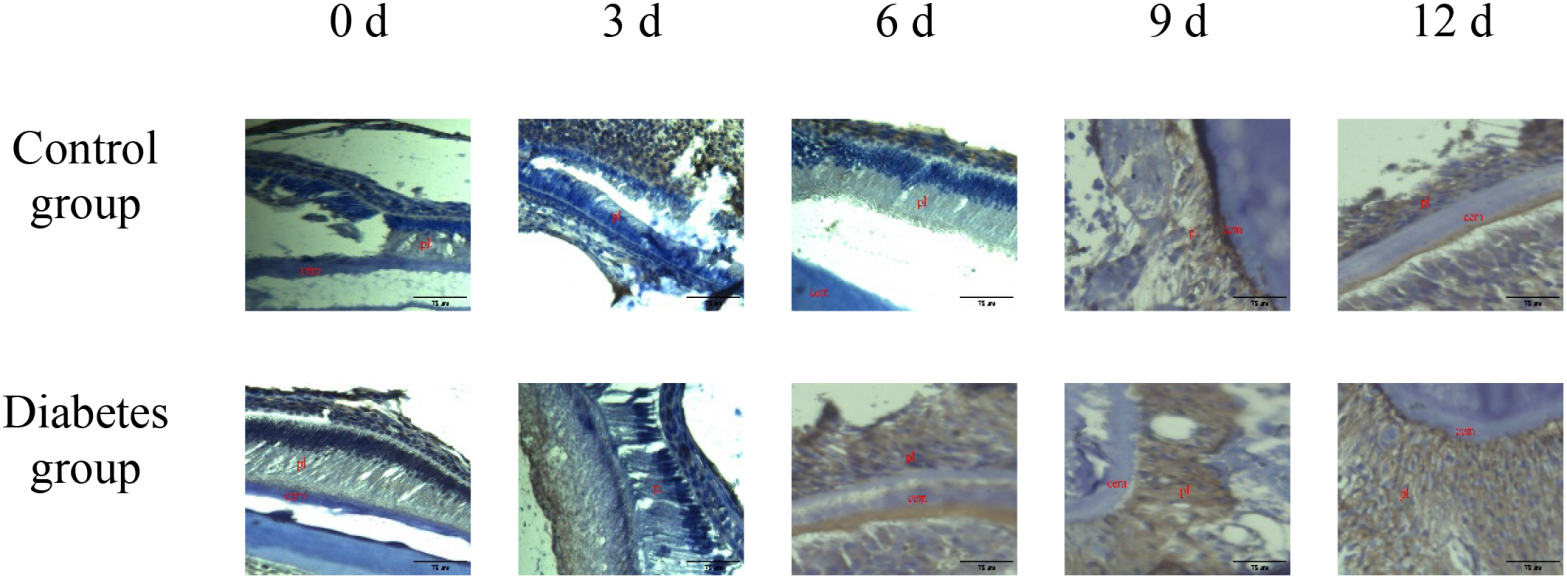
The expression of RANK in periapical tissues of the diabetes group and control group. Immunohistochemical staining showed that RANK protein expression was strongly positive in the diabetes group, and weak positive staining was observed in the control group. cem, cementum; ab, alveolar bone; pl, periodontal ligament (×20).

**Figure 4:**
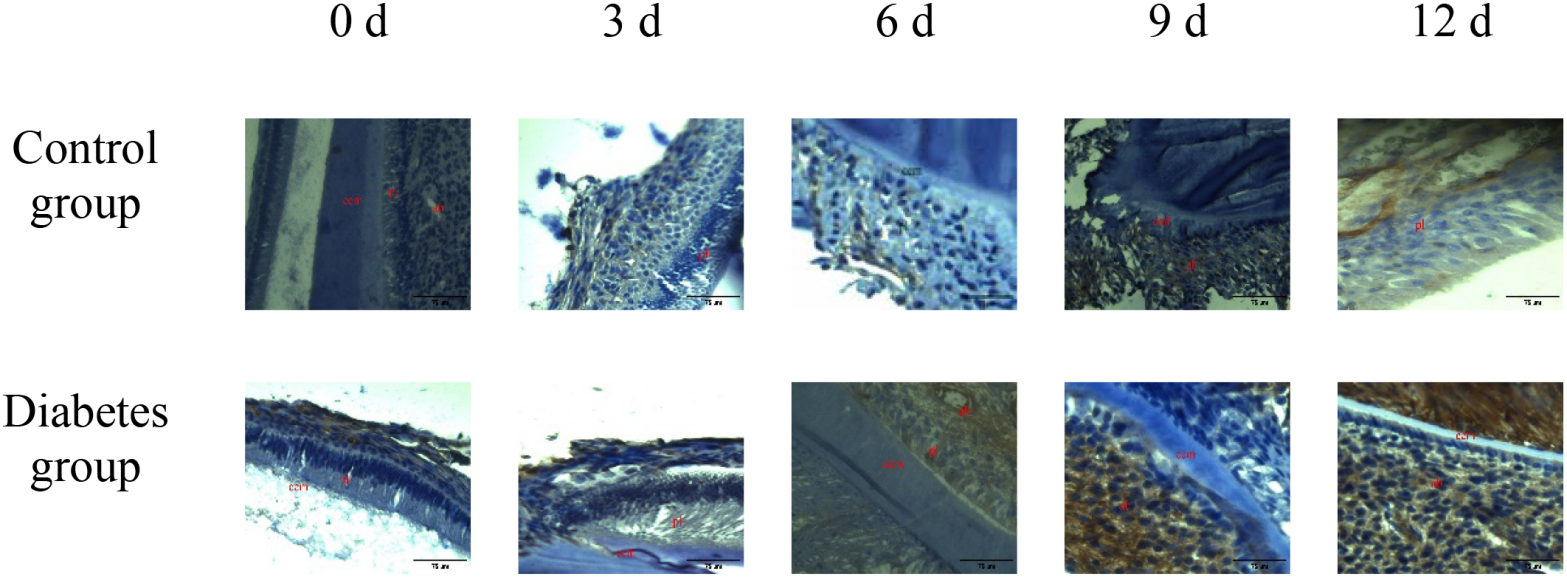
The expression of RANKL in periapical tissues of the diabetes group and control group. Immunohistochemical staining showed that RANKL protein expression was strongly positive in the diabetes group, and weak positive staining was observed in the control group. cem, cementum; ab, alveolar bone; pl, periodontal ligament (×20).

The results of immunohistochemistry showed that RANK, RANKL, and OPN proteins were expressed in the alveolar bone of mandibular molars of the two groups of mice, and with the extension of tooth extraction time, the staining of RANK, RANKL, and OPN degree gradually increased. Compared with the control group, the expression of RANK, RANKL, and OPN increases in the diabetes group, as shown in **Figures 2**–**4**.

**Figure 5:**
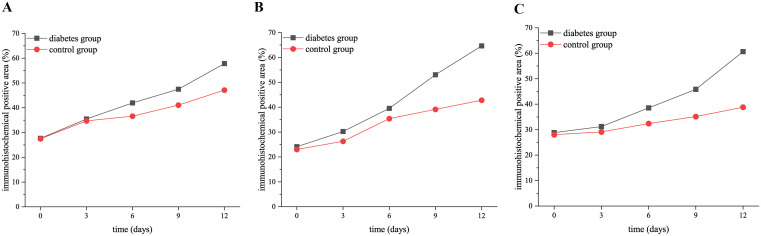
The expression of RANK, RANKL, and OPN proteins in the diabetic group and control group at 0, 3, 6, 9, and 12 days after tooth extraction. **(A)** shows the trend of OPN expression level in both groups at 0, 3, 6, 9, and 12 days after tooth extraction. **(B)** presents the trend of RANK expression level in both groups at 0, 3, 6, 9, and 12 days after tooth extraction. **(C)** shows the trend of RANKL expression level in both groups at 0, 3, 6, 9, and 12 days after tooth extraction. The staining degree of RANK, RANKL, and OPN increased gradually over time. The expression level of OPN, RANK, and RANKL in the diabetes group was significantly higher than that in the control group (*p* < 0.05).

The comparison of positive area percentage showed that the expression level of OPN (0 days: *p* = 0.01, 3 days: *p* = 0.03, 6 days: *p* = 0.01, 9 days: *p* = 0.01, 12 days: *p* < 0.001), RANK (0 days: *p* = 0.03, 3 days: *p* = 0.01, 6 days: *p* = 0.01, 9 days: *p* < 0.001, 12 days: *p* < 0.001), and RANKL (0 days: *p* = 0.01, 3 days: *p* = 0.02, 6 days: *p* = 0.002, 9 days: *p* < 0.001, 12 days: *p* < 0.001) protein in the diabetes group was significantly higher than that in the control group, as shown in **Figure 5**.

#### 3.3.2 Hematoxylin and eosin staining, Masson staining, and tartrate-resistant acid phosphatase activity assay of right mandibles

**Figure 6:**
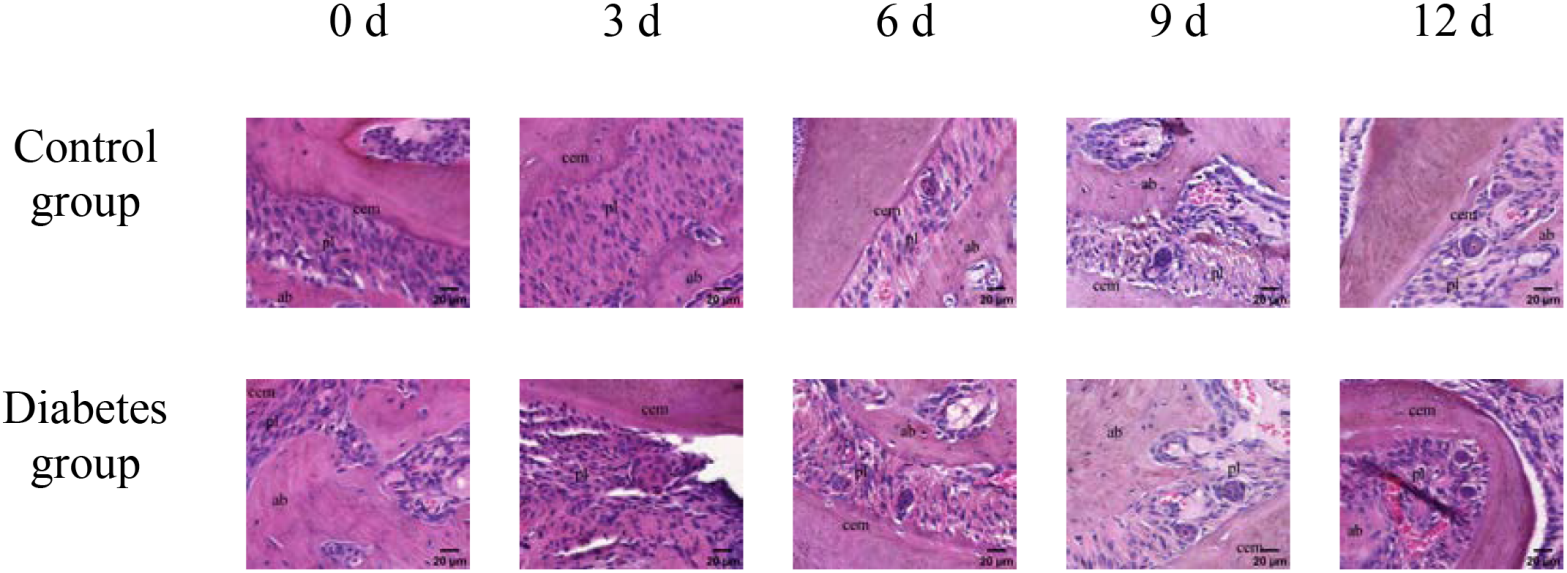
HE staining of right mandibular molar periapical tissue at 0, 3, 6, 9, and 12 days after tooth extraction. From 3 to 12 days after tooth extraction, periodontal ligament was gradually disorganized, cell components around alveolar bone increased, and osteoblasts and osteoclasts could be observed. Compared with the control group, the number of cells in the diabetes group was less, and the number of osteoclasts was significantly more than that in the control group. cem, cementum; ab, alveolar bone; pl, periodontal ligament (×40).

**Figure 7:**
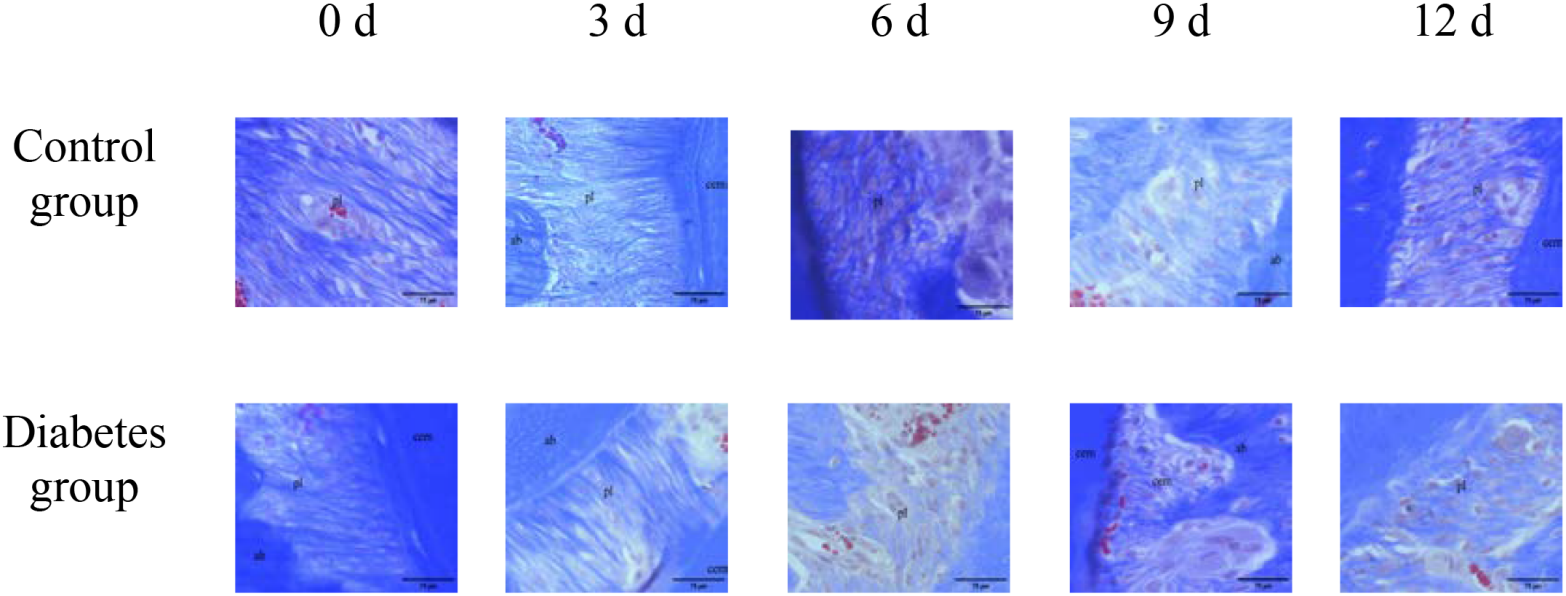
Masson staining of right mandibular molar periapical tissue at 0, 3, 6, 9, and 12 days after tooth extraction. Three to nine days after tooth extraction, the periodontal ligament tissue gradually swelled, and the collagen fibers began to become disordered. Compared with the control group, collagen fibers in the diabetes group were looser and more disordered. cem, cementum; ab, alveolar bone; pl, periodontal ligament (×20). .

**Figure 8:**
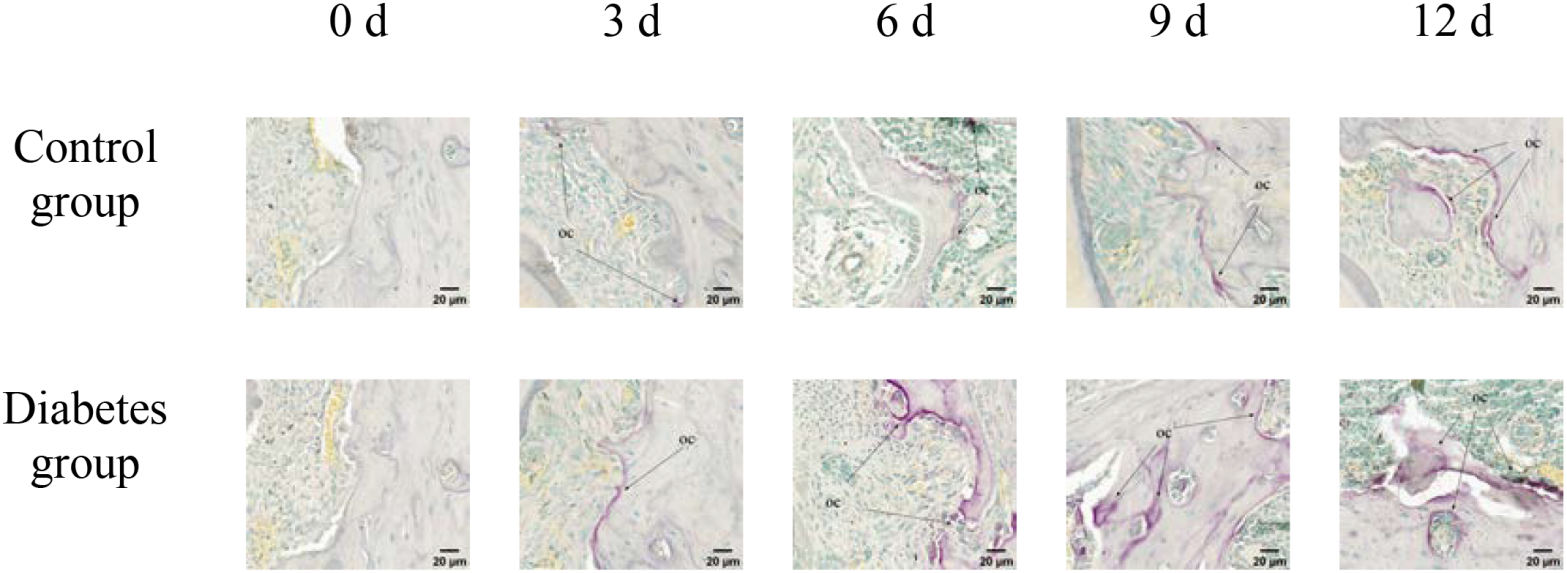
TRAP activity assay of right mandibular molar periapical tissue at 0, 3, 6, 9, and 12 days after tooth extraction. After tooth extraction, the number of osteoclasts gradually increased in both groups, but the number of osteoclasts in the diabetes group was higher than that in the control group. The arrows indicate the site of positive osteoclast staining. OC, osteoclast (×40).

The histological change trends of the two groups at all time points were basically consistent. At 0 days after tooth extraction, the collagen fibers were evenly distributed and arranged neatly, during which there were various cells, and the arrangement direction was basically the same as that of collagen fibers. The edge of alveolar bone was neat and continuous, the trabecula was clear, and no obvious osteoclasts were found. At 3–9 days after tooth extraction, the periodontal ligament tissue gradually swelled, the collagen fibers began to disarrange, the cellular components increased around the alveolar bone, the edge of alveolar bone appeared discontinuous, and osteoclasts appeared. At 12 days after tooth extraction, the destruction range of alveolar bone edges increased, and irregular absorption was visible. Meanwhile, the aggregation of osteoclasts could be seen under the microscope. In the diabetes group, the collagen fibers were sparser and disordered, the bone matrix staining was more uneven, and there were fewer number of cells. The number of osteoclasts was more than that in the control group. Osteoclasts aggregated at 12 days after tooth extraction, and osteoblasts were not obvious, as shown in **Figures 6**–**8**.

### 3.4 Real-time fluorescence quantitative PCR

**Figure 9:**
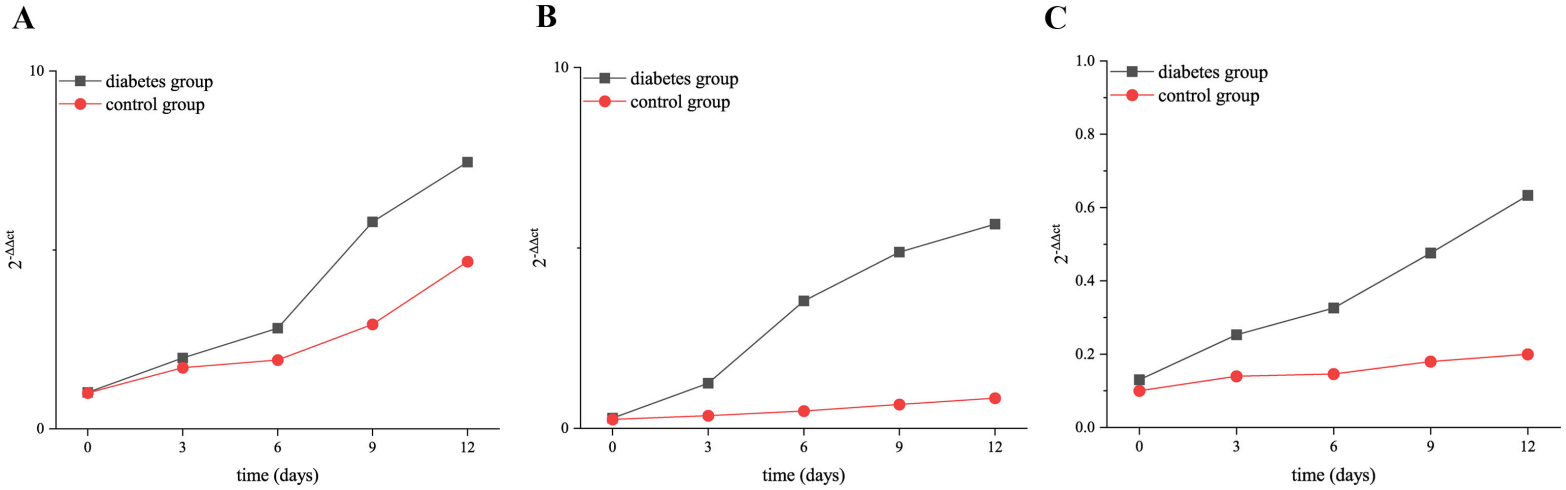
Relative expression of RANK, RANKL, and OPN in the diabetic group and control group at 0, 3, 6, 9, and 12 days after tooth extraction. **(A)** shows the relative expression of OPN in the diabetic group and control group at 0, 3, 6, 9, and 12 days after tooth extraction. **(B)** presents the relative expression of RANK in the diabetic group and control group at 0, 3, 6, 9, and 12 days after tooth extraction. **(C)** shows the relative expression of RANKL in the diabetic group and control group at 0, 3, 6, 9, and 12 days after tooth extraction. The relative expression of RANK, RANKL, and OPN genes increased over time. The relative expression level of OPN, RANK, and RANKL in the diabetes group was significantly higher than that in the control group (*p* < 0.05). *p < 0.05; **p < 0.01; ***p < 0.001.

The results of RT-qPCR showed that the expressions of RANK, RANKL, and OPN genes in the alveolar bone of mandibular molars of the two groups increased with the extension of tooth extraction time. The expression level of OPN (0 days: *p* = 0.03, 3 days: *p* = 0.02, 6 days: *p* = 0.01, 9 days: *p* < 0.001, 12 days: *p* < 0.001), RANK (0 days: *p* = 0.01, 3 days: *p* = 0.01, 6 days: *p* = 0.001, 9 days: *p* < 0.001, 12 days: *p* < 0.001), and RANKL (0 days: *p* = 0.02, 3 days: *p* = 0.01, 6 days: *p* = 0.02, 9 days: *p* = 0.01, 12 days: *p* = 0.004) genes was significantly higher than that of the control group, as shown in **Figure 9**.

## 4 Discussion

Axial tooth movement involves changes in the alveolar bone, which is the result of the interaction between osteoblasts and osteoclasts (11). Presently, research in this area is scant. Diabetes may inhibit alveolar bone remodeling, leading us to conjecture a potential impact on axial tooth movement. Our research indicates that diabetes influences this process (3.50 ± 0.52 mm vs. 4.78 ± 0.55 mm, *p* < 0.001), and it is achieved by inhibiting the expression of osteoclast-related factors RANK, RANKL, and OPN, which affects the growth and development of osteoclasts and promotes the absorption of the alveolar bone (*p* < 0.05).

The global age-standardized prevalence of type 2 diabetes in 2021 was 5.9%, and it may rise to 9.5% by 2050 (25). As the incidence of this condition rises, its impact on oral health has received much attention (26, 27). According to our hypothesis, type 2 diabetes affects axial tooth movement. Therefore, our study established the model of the unopposed molars in type 2 diabetic mice. Micro-CT revealed that the elongation of mandibular molars of the two groups increased gradually with the extension of tooth extraction time, and the elongation of diabetes group was slightly less than that of the control group, suggesting that type 2 diabetes inhibits axial tooth movement.

Tooth movement depends on the resorption and formation of alveolar bone (11). Therefore, we speculate that type 2 diabetes affects tooth movement by affecting the remodeling of alveolar bone. Compared with non-diabetes patients, the risk of fracture in diabetic patients increases by 32%. With the prolongation of the disease time, the risk of fracture also increases gradually (28). Studies have found that the mandibular BMD of type 2 diabetic rats decreased (29); in addition, the resorption of alveolar bone increased, the number of bone trabeculae decreased, and the volume fraction of alveolar bone decreased (30). In this experiment, the mandibular bone density of the diabetic group was lower than that of the control group at 0–12 days after tooth extraction, and the change of alveolar bone of diabetic mice was less than that of the control group at 3–12 days, which was similar to the results of the above study. It is further indicated that diabetes can affect the ability of alveolar bone remodeling in mice.

Hyperglycemia can promote osteoclast differentiation and activation in many ways (17). RANKL is a membrane-associated cytokine, which is mainly expressed on the surface of osteoclasts and their progenitor cells (31). RANK is mainly expressed on the surface of osteoclast progenitor cells and is the only receptor for RANKL. It can recognize and bind RANKL through cell-dependent contact (18). This interaction directly promotes the differentiation, activation, and maturation of osteoclasts; prevents osteoclast apoptosis; and regulates the transcription and expression of related genes (21). The binding of RANK and RANKL is significant for the survival and activation of osteoclasts, positively influencing bone resorption (31). OPN exists in the extracellular matrix (ECM) and has functions of promoting osteoclast migration and differentiation, inhibiting mineral deposition, and accelerating bone resorption (32). Some studies have found (33) that OPN can stimulate the expression of CD44 receptor on the surface of osteoclasts and enhance the activity of osteoclasts (33). A large number of bone remodeling occurs in the periapical tissue of the opposite jaw of missing teeth. Whether the enhanced differentiation of osteoclast in the above process is related to the increased expression of RANK, RANKL, and OPN in a high-glucose environment needs to be further studied.

In this experiment, through immunohistochemical staining and real-time fluorescent quantitative PCR, we found that the expression of RANK, RANKL, and OPN increased over time after tooth extraction in diabetic mice and normal mice, suggesting that RANK, RANKL, and OPN were involved in alveolar bone remodeling. The expression of RANK, RANKL, and OPN in diabetic mice was higher than that in the control group at all time points after tooth extraction, suggesting that diabetes may promote bone resorption by increasing the expression of RANK, RANKL, and OPN. Therefore, it can be speculated that RANK, RANKL, and OPN are involved in alveolar bone remodeling during axial tooth movement, and diabetes mellitus may promote the alveolar bone resorption by increasing the expression of RANK, RANKL, and OPN, leading to a reduction in axial tooth elongation.

Our research employed HE, Masson, and TRAP activity assay, and revealed that diabetic mice demonstrated significant resorption of the alveolar bone margin, accumulation of osteoclasts, and disorganization of periodontal ligament fibers, consistent with the findings of Mei et al. (34). Compared to normal mice, diabetic mice had more pronounced alveolar bone margins and more disorganized periodontal ligament fiber arrangement. There was significant inflammatory cell infiltration in the gingival epithelium, and a part of the gingiva between the molars and gingival epithelium was missing in diabetic mice. This indicates that diabetes can promote osteoclast secretion and affect alveolar bone remodeling, and the specific mechanism needs to be further studied. Combined with the above results of this study, it can be concluded that type 2 diabetes can promote the differentiation of osteoclasts through the abnormal expression of RANK, RANKL, and OPN, affecting the alveolar bone remodeling and thus affecting the axial movement of teeth.

However, this study had several limitations. First, this study investigated the remodeling of the alveolar bone during axial tooth movement in type 2 diabetic mice from the perspective of osteoclast-related factors. However, bone remodeling is the result of the combined actions of osteoclasts and osteoblasts. Therefore, it is necessary to further investigate the mechanisms of alveolar bone remodeling in diabetic mice during tooth axial movement from the perspective of osteoblast-related factors. Second, our study only observed the changes of osteoclast-related factors in the alveolar bone during axial tooth movement in type 2 diabetic mice. In future research, we should examine these factors to further clarify their impact on alveolar bone remodeling.

In summary, the axial movement of teeth in type 2 diabetic mice is significantly reduced. The mechanism may be related to the remodeling of alveolar bone and the action of osteoclast-related factors.

## Data Availability

The original contributions presented in the study are included in the article/supplementary material. Further inquiries can be directed to the corresponding author.
